# Novel Insights Into the Protective Role of Hemoglobin S and C Against *Plasmodium falciparum* Parasitemia

**DOI:** 10.1093/infdis/jiv098

**Published:** 2015-02-23

**Authors:** Valentina D. Mangano, Youssouf Kabore, Edith C. Bougouma, Federica Verra, Nuno Sepulveda, Cyrille Bisseye, Federica Santolamazza, Pamela Avellino, Alfred B. Tiono, Amidou Diarra, Issa Nebie, Kirk A. Rockett, Sodiomon B. Sirima, David Modiano

**Affiliations:** 1 Department of Public Health and Infectious Diseases; 2 Istituto Pasteur–Fondazione Cenci Bolognetti, Sapienza University of Rome, Italy; 3 Centre National de Recherche et Formation sur le Paludisme, Ouagadougou, Burkina Faso; 4 London School of Hygiene and Tropical Medicine; 5 Wellcome Trust Centre for Human Genetics, University of Oxford; 6 Wellcome Trust Sanger Institute, Hinxton, Cambridge, United Kingdom; 7 Center of Statistics and Applications of University of Lisbon, Portugal

**Keywords:** Burkina Faso, Fulani, hemoglobin C, hemoglobin S, *Plasmodium falciparum*, infection

## Abstract

Although hemoglobin S (HbS) and hemoglobin C (HbC) are well known to protect against severe *Plasmodium falciparum* malaria, conclusive evidence on their role against infection has not yet been obtained. Here we show, in 2 populations from Burkina Faso (2007–2008), that HbS is associated with a 70% reduction of harboring *P. falciparum* parasitemia at the heterozygous state (odds ratio [OR] for AS vs AA, 0.27; 95% confidence interval [CI], .11–.66; *P* = .004). There is no evidence of protection for HbC in the heterozygous state (OR for AC vs AA, 1.49; 95% CI, .69–3.21; *P* = .31), whereas protection even higher than that observed with AS is observed in the homozygous and double heterozygous states (OR for CC + SC vs AA, 0.04; 95% CI, .01–.29; *P* = .002). The abnormal display of parasite-adhesive molecules on the surface of HbS and HbC infected erythrocytes, disrupting the pathogenic process of sequestration, might displace the parasite from the deep to the peripheral circulation, promoting its elimination at the spleen level.

Sickle hemoglobin (hemoglobin S [HbS]) results from a single-nucleotide polymorphism (SNP; rs334) of the *HBB* gene encoding the β-globin chain, leading to an aminoacidic substitution from glutamic acid to valine (sixth codon: GAG → GTG). Individuals with the SS genotype have sickle cell anemia, a highly lethal condition [1].

Nevertheless, HbS maintains a high frequency in sub-Saharan Africa, probably owing to the survival advantage against malaria of individuals with the AS genotype. There is much epidemiological evidence that HbS protects against severe [2–8] and uncomplicated [9–12] *Plasmodium falciparum* malaria in heterozygosis. Furthermore, multiple studies have shown lower parasite densities during symptomatic malaria in subjects with the AS genotype than for those with AA [13–15]. Similar evidence is not available for protection against infection. A systematic review [16] pointed out that the role of the AS genotype on *P. falciparum* infection has been investigated mainly through single cross-sectional surveys of the prevalence of parasitemia, yielding conflicting results. Some studies found reduced prevalence in subjects with AS compared with AA [17–20], but others showed a similar [21–30] or even an increased prevalence [31, 32]. The review [16] found only 2 longitudinal studies of the incidence of parasitemia [33, 34], reporting similar rates in subjects with AA and those with AS. More recently, however, a longitudinal study conducted in Uganda showed that children with the AS genotype had a lower number of new strains detected per person-year than those with the AA genotype and that the effect of HbS was greater in older children [35]. A second longitudinal study conducted in Mali showed that children with the AS genotype remained smear-negative for a longer time than those with the AA genotype [36].

Hemoglobin C (HbC) results from a SNP (rs33930165) of the *HBB* gene leading to an aminoacidic substitution from glutamic acid to lysine (sixth codon: GAG → AAG). Individuals with the AC genotype are asymptomatic, those with the CC genotype have mild hemolytic anemia, and SC double heterozygotes have moderate sickle cell disease [1].

HbC is known to protect against severe malaria in an additive way, with CC homozygotes showing a much higher degree of protection than subjects with the AC genotype [2, 3, 6, 8]. There is not as much evidence of protection conferred by HbC against uncomplicated malaria: CC homozygotes shows protection in 1 study [2], and subjects with AC show protection in case-control [2, 37] but not perspective studies [11, 12]. The role of HbC in parasitemia has been addressed by 6 cross-sectional studies [21, 23, 25, 31, 38] and 1 longitudinal study [12], none of which provided evidence for protection in subjects with the AC genotype [16]. A reduced prevalence of *P. falciparum* infection was observed in subjects with CC or SC genotypes in only 1 cross-sectional study conducted in Ghana [25], but differences did not reach statistical significance.

Our study aimed to provide new insights into the protective role of HbS and HbC against *P. falciparum* infection. We conducted 5 cross-sectional surveys in rural villages of Burkina Faso inhabited by the Fulani, Mossi, and Rimaibe communities. The Fulani were reported elsewhere to be less infected with *P. falciparum* and to have a different genetic background from their neighbors, whereas the Mossi and Rimaibe show comparable susceptibility to infection, are genetically similar [39, 40], and will be thus grouped together as *non-Fulani*.

## METHODS

### Ethics Statement

The study received approval from the ethical committees of the Ministry of Health of Burkina Faso and the University of Oxford. Study subjects or their guardians gave written informed consent for participation.

### Study Area and Populations

The study was carried out in 4 rural villages of Burkina Faso, northeast (Barkoumbilen and Barkoundouba) and east (Bassy and Zanga) of Ouagadougou. Malaria transmission is hyperendemic and seasonal, with a rainy season from June to October. The entomological inoculation rates, estimated at about 100–200 infective bites per person per year, are similar across villages [39].

### Study Design and Epidemiological Surveys

The study had a repeated cross-sectional design: 5 surveys were carried out, at the beginning (August) and end (November/December) of the 2007 and 2008 high malaria transmission seasons and in the middle of the intervening dry low transmission season (March 2008). For each survey a team of physicians examined participants for clinical signs, measured axillary body temperature, and prepared blood slides for malaria diagnosis from finger pricks. Subjects exhibiting fever (temperature, ≥37.5°C) were treated presumptively with artemether-lumefantrine (Coartem) according to manufacturer's dosage recommendations. No treatment was offered to carriers of asymptomatic parasitemia. During the first survey when a subject entered the study, a 2-mL venous blood sample was collected in ethylenediaminetetraacetic acid tubes for DNA extraction.

### Parasitological Diagnosis

*Plasmodium falciparum* asexual parasitemia was microscopically diagnosed. Blood slides with thick and thin blood smears were prepared and stained with Giemsa stain according to standard procedures and read independently by 2 skilled microscopists. The *Plasmodium* species was identified on the thin blood smear. Readers examined 100 microscopic fields (corresponding to 0.25 µL of blood) from the thick blood smear, parasite counts were converted to numbers of parasites per microliter of blood (assuming a standard count of 8000/μL), and the mean density from 2 readings was used. A third reader was involved when the 2 readers disagreed about positivity or when estimated densities differed by >30%. In these cases, the mean of the 2 closest density readings was used.

Microscopic diagnosis was confirmed on DNA samples by polymerase chain reaction amplification of *P. falciparum EBA175* and *TRAP* sequences (Supplementary Methods). Across all surveys, *P. falciparum* accounted for 91.4% of malaria infections, followed by *P. malariae* (2.1%) and *P. ovale* (0.2%); 6.3% were mixed infections. Of the *P. falciparum* infections, 4.5% were symptomatic (parasitemia associated with fever).

### DNA Extraction and *HBB* Genotyping

Genomic DNA was extracted from whole blood using the Nucleon BACC2 Kit. The rs334 (A → HbA/T → HbS) and rs33930165 (G → HbA /A → HbC) SNPs at the *HBB* locus were genotyped using the Sequenom MassArray System [41]. Genotypes at the 2 SNPs were combined to obtain the *HBB* genotypes: HbAA (AA + GG), HbAC (AA + AG), HbAS (AT + GG), HbCC (AA + AA), HbSC (AT + AG), and HbSS (TT + GG). Polymerase chain reaction [42] and sequencing (BMR Genomics) were used to validate genotype calls for the rare genotypes CC, SC, and SS (expected frequency, <1%). The genotyping assays had 97% and 98% success rates respectively, resulting in a complete *HBB* genotype for 95% of recruited subjects. Genotypes were in Hardy–Weinberg equilibrium within each population.

### Statistical Analysis

Statistical analysis was conducted both at the single survey level as well as longitudinally, and outcome data were compared between subjects with AC, AS, CC, SC, and AA (reference) genotypes. At each of the 5 cross-sectional surveys (n = 1162, n = 1595, n = 1281, n = 1554, and n = 1524, respectively), we compared the prevalence of asymptomatic parasitemia. In longitudinal analyses we included all infection events, regardless of whether or not parasitemia was associated with fever. Among subjects who participated in all surveys (n = 481), we compared the proportion infected at least once, the total number of infections, and the mean parasite density over 5 measurements. Finally, starting from the first survey (n = 1162), we compared the proportion of subjects who remained uninfected over the 5 measurements.

Prevalences were compared using a maximum likelihood estimate of the odds ratios (ORs) and 95% confidence intervals (CIs) within each ethnic group and a Mantel–Haenszel χ^2^ test stratifying by ethnicity in the overall population. The proportions of individuals infected at least once were compared using logistic regression, the numbers of infections using Poisson regression, and the mean parasite densities using linear regression after logarithmic transformation. The proportions of subjects who remain uninfected over time were compared using survival analysis based on lifetime tables and log-rank test for the equality of survival functions, and the cumulative probabilities of infection were compared using Poisson regression. All regression models included age (in years) as a covariate. Ethnicity (Fulani or non-Fulani group) was included as a covariate when the analysis was conducted in the overall population. Two-sided *P* values were reported, with differences considered significant at *P* ≤ .05. All analyses were carried out with the statistical software package Stata/IC 10.0 (StataCorp LP, College Station, Texas).

## RESULTS

### Frequencies of HbS and HbC

The study included a total of 2206 subjects. The frequencies of HbS and HbC (Table 1) are consistent with previous data in the study populations [2, 43]. The frequency of HbS was 4%, irrespectively of ethnicity, sex, or age group, and the frequency of HbC was lower in Fulani (5%) than in non-Fulani (12%), as reported elsewhere [43]. Because ethnicity [39] and age [44] play a major role in immunity to infection, we adjusted all genotype-phenotype association analyses for these 2 factors.

**Table 1.** Characteristics of Study Subjects and Frequency of HbS and HbC

Factor	Subjects by *HBB* Genotype, No.						Frequency (95% CI)	
	AA	AC	AS	CC	SC	Total	HbS	HbC
Ethnicity								
Fulani	708	80	80	0	2	870	0.05 (.03–.06)	0.05 (.03–.06)
Non-Fulani	930	282	98	15	11	1336	0.04 (.03–.05)	0.12 (.10–.14)
Sex								
Male	722	166	84	6	6	984	0.05 (.03–.06)	0.09 (.08–.11)
Female	916	196	94	9	7	1222	0.04 (.03–.05)	0.09 (.07–.11)
Age group, y^a^								
≤5	321	71	30	4	1	427	0.04 (.02–.06)	0.09 (.07–.12)
>5 ≤ 10	319	64	27	3	2	415	0.03 (.02–.05)	0.09 (.06–.11)
>10 ≤ 20	449	100	53	4	6	612	0.05 (.03–.07)	0.09 (.07–.12)
>20	549	127	68	4	4	752	0.05 (.03–.06)	0.09 (.07–.11)
Total	1638	362	178	15	13	2206	0.04 (.04–.05)	0.09 (.08–.10)

Abbreviations: CI, confidence interval; HbC, hemoglobin C; HbS, hemoglobin S.

^a^ Based on age at the first survey.

### Proportion of *P. falciparum*–Positive Slides

During 5 surveys conducted from August 2007 to December 2008, we performed 7008 microscopic diagnoses of malaria in asymptomatic subjects (2792 in Fulani and 4216 in non-Fulani subjects). The percentage of *P. falciparum*–positive slides varied according to *HBB* genotype: compared with that for subjects with the AA genotype, percentages were lower for subjects with the AS, CC, or SC genotype, in both ethnic groups, whereas for subjects with the AC genotype the percentage was similar (non-Fulani) or even higher (Fulani) than for AA (Table 2).

**Table 2.** Proportion of *Plasmodium falciparum*–Positive Blood Slides According to *HBB* Genotype

*HBB* Genotype by Ethnicity	Subjects, No.			*P. falciparum* Positive, %
	*P. falciparum* Negative	*P. falciparum* Positive	Total	
Fulani				
AA	1734	530	2264	23.4
AC	183	76	259	29.3
AS	209	55	264	20.8
SC	4	1	5	20.0
Total	2130	662	2792	23.7
Non-Fulani				
AA	1566	1386	2952	47.0
AC	489	425	914	46.5
AS	159	103	262	39.3
CC	29	20	49	40.8
SC	30	9	39	23.1
Total	2273	1943	4216	46.1

### Cross-sectional Analysis: Prevalence of Asymptomatic *P. falciparum* Parasitemia

We therefore stratified the sample by survey to investigate the association of HbS and HbC with the prevalence of *P. falciparum* asymptomatic infection. The results of the statistical comparison among *HBB* genotypes are shown in Table 3. The data do not convey any consistent pattern of association with prevalence of infection across surveys or populations. Indeed, in both Fulani and non-Fulani groups, the AC genotype, compared with the wild-type AA genotype, shows similar prevalences (OR ≈ 1) in some surveys and even higher prevalences (OR > 1) in others (difference close to significance in the overall population for the fourth survey). On the contrary, in both populations the AS genotype shows similar prevalences in some surveys but lower prevalences (OR < 1) in others (significant difference in the overall population for the first and fifth surveys). The CC genotype is present only in the non-Fulani group and shows similar prevalences in some surveys and lower prevalences (OR << 1) in others, although the difference never reached statistical significance. Finally, for surveys in which comparison was possible in both populations, the SC genotype showed lower prevalences (OR << 1) than the AA genotype (significant difference in the overall population for the fourth survey).

**Table 3.** Statistical Comparison of the Prevalence of *P. falciparum* Asymptomatic Infection According to *HBB* Genotype at Each of 5 Cross-sectional Surveys

*HBB* Genotype by Survey	Fulani		Non-Fulani		Overall	
	OR (95% CI)	*P* Value^a^	OR (95% CI)	*P* Value^a^	OR (95% CI)	*P* Value^b^
AC						
1	1.06 (.58–1.92)	.85	0.87 (.61–1.23)	.43	0.91 (.68–1.24)	.56
2	1.44 (.79–2.62)	.23	0.90 (.65–1.24)	.52	0.99 (.75–1.32)	.97
3	1.27 (.56–2.84)	.56	0.83 (.54–1.27)	.39	0.90 (.62–1.31)	.59
4	1.73 (.94–3.20)	.94	1.23 (.89–1.71)	.89	1.32 (.99–1.76)	.06
5	1.15 (.58–2.27)	.70	1.03 (.75–1.42)	.87	1.05 (.78–1.40)	.75
AS						
1	0.48 (.21–1.09)	.07	0.51 (.26–1.00)	.05	0.50 (.29–.84)	.01
2	0.87 (.47–1.61)	.66	1.13 (.68–1.87)	.63	1.02 (.69–1.50)	.93
3	1.33 (.59–2.99)	.49	1.03 (1.53–1.99)	.93	1.14 (.68–1.89)	.63
4	1.12 (.61–2.08)	.71	0.64 (.36–1.13)	.12	0.83 (.54–1.25)	.37
5	0.85 (.43–1.70)	.65	0.52 (.29–.95)	.03	0.64 (.41–1.01)	.05
CC						
1	…	…	1.17 (.19–7.06)	.86	…	…
2	…	…	0.45 (.14–1.47)	.17	…	…
3	…	…	1.13 (.29–4.32)	.86	…	…
4	…	…	1.18 (.37–3.75)	.78	…	…
5	…	…	0.57 (.14–2.30)	.42	…	…
SC						
1	1.61 (.10–26.17)	.73	0.19 (.02–1.77)	.10	0.40 (.09–1.88)	.23
2	…		0.50 (.12–2.03)	.33	…	…
3	…		1.00 (.20–5.04)	>.99	…	…
4	0.00 (Nc)	.55	0.09 (.01–.75)	.01	0.09 (.01–.73)	.00
5	0.00 (Nc)	.46	0.46 (.09–2.37)	.34	0.39 (.07–2.02)	.24

“…” indicates there were no CC subjects in the Fulani ethnic group.

Abbreviations: CI, confidence interval; Nc, not computed; OR, odds ratio.

^a^ Maximum likelihood estimation of the OR.

^b^ *P* values determined with Mantel–Haenszel χ^2^ test.

### Longitudinal Analysis of Repeated Cross-sectional Data: Odds of *P. falciparum* Infection, Number of Infections, and Mean Parasite Counts Over 5 Measurements

The prevalence of *P. falciparum* parasitemia at a given moment in a given population can vary with the measurement itself as well as with many environmental (eg, transmission level) or parasite (eg, strains with different abilities to establish infection) factors other than host factors. Therefore, it might not be the most appropriate phenotype to evaluate the role of human genetic variants in protection from infection, which is why we used what we believe to be a more robust phenotype indicating susceptibility/resistance: the odds of being infected with *P. falciparum* at least once during 5 surveys.

We included in the analysis individuals included in all 5 surveys (n = 481). We defined as not infected those subjects who never had a diagnosis of *P. falciparum* parasitemia, and as infected those who had *P. falciparum* parasitemia diagnosed at least once (Table 4). In both the Fulani (n = 186) and non-Fulani (n = 295) groups, we observed that the odds of infection for subjects with the AC genotype is comparable to that for those with AA (OR in the overall population, 1.49; 95% CI, .69–3.21; *P* = .31). This is not the case for subjects with AS, who showed much lower odds than those with AA in both ethnic groups. In the overall population, with adjustment for ethnicity and age, we indeed observed a 70% reduction in the odds of infection for carriers of HbS (OR, 0.27; 95% CI, .11–.66; *P* = .004).

**Table 4.** Proportion of Subjects Infected With *Plasmodium falciparum* at Least Once Over 5 Surveys According to *HBB* Genotype and Statistical Comparison

*HBB* Genotype by Ethnicity	Subjects, No.^a^			OR (95% CI)^b^	*P* Value^c^
	*P. falciparum* Negative	*P. falciparum* Positive	*P. falciparum* Positive , %		
Fulani					
AA	49	97	66.4	…	…
AC	9	14	60.9	1.13 (.36–3.58)	.83
AS	11	6	35.3	0.32 (.09–1.06)	.06
Non-Fulani					
AA	21	181	89.6	…	…
AC	6	63	91.3	1.8 (.62–5.22)	.28
AS	6	12	66.7	0.24 (.07–.82)	.02
CC	1	1	50	0.11 (.00–3.40)	.21
SC	2	1	33.3	0.02 (.00–.28)	.003

Abbreviations: CI, confidence interval; OR, odds ratio.

^a^ The *P. falciparum*–negative group includes subjects not infected with *P. falciparum* over 5 cross-sectional surveys; the *P. falciparum*–positive group, subjects infected with *P. falciparum* at least once over 5 cross-sectional surveys.

^b^ ORs were adjusted for age group and ethnicity.

^c^ *P* values based on logistic regression for the statistical comparison of odds, where the wild-type AA genotype is used as the reference group.

In the non-Fulani group, we could also observe that individuals with CC or SC genotypes show lower odds of infection than those with AA, although the comparison reaches statistical significance only for SC. It must be noted that these genotype groups are very small. If we combine CC and SC genotypes in a unique group of individuals with double-mutant hemoglobin, these show >90% reduction in the odds of infection (OR for CC + SC vs AA, 0.04; 95% CI, .01–.29; *P* = .002). It seems therefore that HbS is sufficient in single copy to protect from infection, whereas HbC must be associated with either another copy of HbC or with HbS for protection to be achieved. This is similar to what was observed with respect to the protection conferred against severe malaria, where HbC has an additive effect, and individuals with the CC genotype show a much higher degree of protection than those with AC [2].

It is also interesting to note that the protection seen with AS, CC, and SC genotypes in the non-Fulani group brings the odds of infection to levels comparable to that of individuals with wild-type AA in the Fulani group. The observation that the odds of infection is lower in the Fulani for any *HBB* genotype, together with the observation that HbS and HbC are present at comparable and lower frequencies, respectively, further confirms that mutant hemoglobins are not responsible for the lower susceptibility to malaria observed in this ethnic group [44]. Comparable results were obtained applying a standard repeated measure approach, using data from the 5 surveys and a mixed logistic regression model including a random effect describing the statistical dependency between measurements from the same individual (Supplementary Table 1).

In the same group of subjects, we also looked at the number of infections measured over 5 surveys according to *HBB* genotype (data not shown). We observed that the numbers of infections are comparable for subjects with AC and those with AA in both Fulani and non-Fulani groups and in the overall population (incidence rate ratio [IRR], 1.08; 95% CI, .92–1.27; *P* = .33). However, the numbers of infections are lower for AS than for AA in both ethnic groups, although this difference reached statistical significance only in the overall population (IRR, 0.72; 95% CI, .53–.98; *P* = .04). In the non-Fulani group, there was a nonsignificant reduction in the number of infections with the CC (IRR, 0.46; 95% CI, .11–1.85; *P* = .27) and SC (IRR, 0.48; 95% CI, .18–1.28; *P* = .14) genotypes and also when the 2 genotypes are grouped (IRR, 0.47; 95% CI, .21–1.06; *P* = .07).

Finally, we compared the mean parasite density observed during the 5 surveys (ie, the mean of 5 trophozoite counts) among *HBB* genotypes (data not shown). In both study populations, no difference in mean parasite density (log-transformed parasite count per microliter) was observed between AC and AA genotypes (OR, 1.07; 95% CI, .91–1.25; *P* = .42 in the overall population). A nonsignificant reduction in mean parasite density was observed in both Fulani and non-Fulani groups when comparing AS and AA genotypes, and this difference reached statistical significance in the overall population (OR, 0.78; 95% CI, .62–.99; *P* = .04). A reduction was observed among the non-Fulani for the CC (OR, 0.48; 95% CI, .18–1.27; *P* = .14) and SC (OR, 0.39; 95% CI, .18–.87; *P* = .02) genotypes, although the difference was significant only for SC. A significant protective effect was observed in the combined group of double-mutant individuals (OR, 0.43; 95% CI, .23–.79; *P* = .007).

### Survival Analysis of Repeated Cross-sectional Data: Proportion of Subjects Who Remain *P. falciparum* Negative

We finally investigated whether HbS and HbC affect the proportion of subjects who remain uninfected over surveys. We included in this analysis all individuals present at the first survey (n = 1163) and followed them up until the end of the study, looking at the first time they became infected or whether they stayed clear of infection.

**Figure 1.**
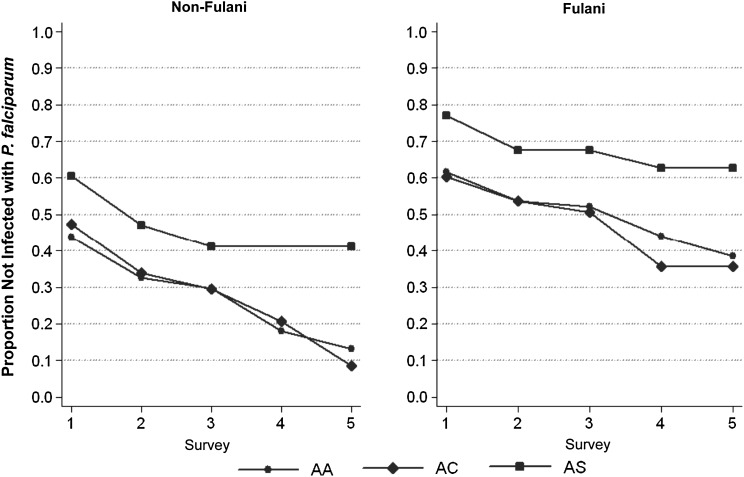
Lifetime curves showing proportions of individuals who remain uninfected with *Plasmodium falciparum* across 5 cross-sectional surveys, according to *HBB* genotype; results are displayed separately for Fulani (*right*) and non-Fulani (*left*) groups.

The lifetime table resulting from this analysis is shown in Supplementary Table 2. Because only the first survey contributed information for subjects with CC and SC genotypes, we excluded these individuals from statistical analysis. Survival curves for subjects AA, AC, and AS genotypes are shown in Figure 1, and the results of the log-rank test in Table 5. In both Fulani (n = 401) and non-Fulani (n = 761) groups, we observed that the proportion of individuals with the AC genotype who remain uninfected is always comparable to that of those with AA (hazard ratio, 1.02; 95% CI, .86–1.23; *P* = .75 in the overall population with adjustment for ethnicity and age). On the contrary the proportion of uninfected individuals is always higher for AS than for AA. In the overall population, subjects carrying the HbS mutation show a lower cumulative probability of becoming infected (hazard ratio, 0.68; 95% CI, .47–.97; *P* = .03 with adjustment for ethnicity and age). Similar results have been obtained in longitudinal studies conducted in Uganda [35] and Mali [36].

**Table 5.** Statistical Comparison of Survival Curves According to *HBB* Genotype

*HBB* Genotype by Ethnicity	Infections Observed, No.	Infections Expected, No.	*P* Value^a^
Fulani			
AA	159	152.1	…
AC	29	29.2	.66
AS	12	18.9	.047
Non-Fulani			
AA	391	381.8	…
AC	121	121.6	.93
AS	20	29.2	.02

^a^ *P* values were determined with the log-rank test for equality of survival curves, with the wild-type AA genotype used as the reference group.

### Modification of HbS Protective Effect by Age Group and Ethnicity

Gong and colleagues [35] observed in their study that the protective effect of HbS increased with age. We therefore stratified our sample into subjects aged ≤10 years and those aged >10 years to investigate whether age modifies the effect of HbS on the odds of infection, number of infections, mean parasite count, and cumulative probability of infection (Supplementary Table 3). For each phenotype analyzed, we observed no protective effect of HbS in the younger group but a significant effect in the older group. We also observed that in subjects aged >10 years the protective effect of HbS (except with respect to mean parasite counts) seemed greater in the Fulani than in the non-Fulani group, although there is no statistical evidence of effect modification (data not shown).

## DISCUSSION

This study was intended to elucidate the role of HbS and HbC in susceptibility to *P. falciparum* infection. One important challenge encountered in such studies is defining an infection phenotype for association analysis. Indeed, the point prevalence of *P. falciparum* parasitemia might not be a robust phenotype, because it can vary with both measurement and epidemiological factors. Other phenotypes might prove more useful, such as the odds of infection, the number of infections, the mean parasite density over repeated measurements, and the proportion of subject who remain clear of infection over time. These can be based on a series of cross-sectional surveys, as in our study, or, better, from cohort studies.

We actually observed that individuals who are heterozygotes for HbS show lower prevalences of parasitemia than AA homozygotes in some but not every survey, and not always in both study populations. This observation reflects discrepancies among results of studies based on single cross-sectional surveys [16]. In longitudinal analysis, however, AS heterozygotes show lower odds of infection than AA homozygotes, fewer infections, lower mean parasite density, and a higher proportion of subjects who remain clear of infection over time, in both Fulani and non-Fulani groups, suggesting a protective role of HbS against the establishment of *P. falciparum* parasitemia. These results are consistent with those obtained by Billo and colleagues [36], who observed that a protective role of HbS against infection could be observed only when the data were analyzed longitudinally and not when the same data were analyzed using a cross-sectional approach.

By stratifying the sample by age group, we were able to observe that the protective effect of HbS becomes apparent in subjects aged >10 years, consistently with a recent report by Gong and colleagues [35]. Furthermore, this protective effect is greater in the less susceptible and more immune reactive Fulani population [39, 45]. These 2 observations suggest that the role of HbS is magnified by concomitant immunity of the host. The fact that the effect of HbS is greater at older ages could also indicate, as suggested elsewhere [35], that the mechanism of protection involves acquired immunity. However, because the Fulani are less susceptible to infection on a different genetic basis than HbS, the fact that the effect of HbS is greater in this ethnic group suggests that the mechanism of protection is amplified by, and not only mediated by, the host immune response, either innate or acquired.

With respect to HbC, our data do not provide evidence for protection against any of the phenotypes analyzed at the heterozygote state. Nevertheless, they suggest a protective effect of HbC in a double-mutant state, that is, in CC homozygotes and SC double heterozygotes. The level of protection observed is similar if not higher to that observed in subjects with the AS genotype. However, given the low frequencies of these genotypes (<1%), our study sample size is too small to draw solid conclusions.

Our results suggest a scenario made of consistent experimental and epidemiological observations. The abnormal display of parasite-adhesive molecules (ie, PfEMP1) on the surface of the HbS and HbC infected erythrocytes [46, 47], disrupting the pathogenic process of sequestration, might displace the parasite from the deep to the peripheral circulation, hence promoting its elimination at the spleen level. It has been proposed [48] that the increased phagocytosis of the HbS and HbC infected erythrocytes in the spleen may also result in improved antigen presentation, which could explain an increase of the protective effect against infection with age, as well as the higher immune response to variant surface antigens observed in individuals carrying those hemoglobinopathies [13, 49, 50]. A protective role of HbS and HbC against infection per se is coherent with the observation that both mutant hemoglobins protect against all syndromes of severe malaria (namely, cerebral malaria and severe malarial anemia) across diverse populations [8, 16].

## Supplementary Data

Supplementary materials are available at *The Journal of Infectious Diseases* online (http://jid.oxfordjournals.org). Supplementary materials consist of data provided by the author that are published to benefit the reader. The posted materials are not copyedited. The contents of all supplementary data are the sole responsibility of the authors. Questions or messages regarding errors should be addressed to the author.

Supplementary Data
