# Supplementary material for: Novel Insights Into the Protective Role of Hemoglobin S and C Against Plasmodium falciparum Parasitemia
Source: J Infect Dis. 2015 Feb 23;212(4):626–34. doi: 10.1093/infdis/jiv098 (PMC4512610; doi:10.1093/infdis/jiv098)
Supplement: Supplementary Data [file supp_212_4_626__index.html]

Novel insights into the protective role of haemoglobin S and C against Plasmodium falciparum parasitaemia — Novel Insights Into the Protective Role of Hemoglobin S and C Against Plasmodium falciparum Parasitemia — Novel Insights Into the Protective Role of Hemoglobin S and C Against Plasmodium falciparum Parasitemia — Supplementary Data 

# Novel Insights Into the Protective Role of Hemoglobin S and C Against *Plasmodium falciparum* Parasitemia

## Supplementary Data

Supplementary Data

**Files in this Data Supplement:**

- Supplementary Data - Doc file
