# Supplementary material for: Novel Insights Into the Protective Role of Hemoglobin S and C Against Plasmodium falciparum Parasitemia
Source: J Infect Dis. 2015 Feb 23;212(4):626–34. doi: 10.1093/infdis/jiv098 (PMC4512610; doi:10.1093/infdis/jiv098)
Supplement: Supplementary Data [file supp_jiv098_jiv098supp.doc]

**SUPPLEMENTARY MATERIAL**

**Table S1**. Results of association analysis of *HBB* genotypes with *P.* *falciparum* asymptomatic parasitaemia (yes/no) at five cross-sectional surveys, Burkina Faso, 2007-8.

| ***HBB*** | **OR** | **95% CI** | ***P*** |
| --- | --- | --- | --- |
| **Fulani** | | | |
| AA | - | - | - |
| AC | 1.21 | 0.60, 2.41 | 0.596 |
| AS | 0.38 | 0.15, 0.98 | 0.044 |
| **Non-Fulani** | | | |
| AA | - | - | - |
| AC | 1.16 | 0.83, 1.64 | 0.385 |
| AS | 0.56 | 0.30, 1.05 | 0.072 |
| CC | 0.22 | 0.03, 1.78 | 0.158 |
| SC | 0.21 | 0.05, 0.98 | 0.046 |
| **Overall** | | | |
| AC | 1.17 | 0.86, 1.60 | 0.318 |
| AS | 0.49 | 0.29, 0.83 | 0.007 |

Abbreviations: OR, Odds Ratio; CI, Confidence Interval; *P*, P-value.

To analyse data on *P. falciparum* asexual parasitaemia (yes/no) from five cross-sectional surveys, we adopted a mixed logistic regression approach using the *lme4* package (Douglas Bates, Martin Maechler, Ben Bolker and Steven Walker 2013. lme4: Linear mixed-effects models using Eigen and S4. R package version 1.0-4. http://CRAN.R-project.org/package=lme4) available for the R software (http://www.r-project.org). In this approach, we fitted a logistic model that includes fixed effects of *HBB* genotypes, age (years), and ethnicity (Fulani and Non-Fulani) together with a random effect describing the statistical dependency between measurements from the same individual. The significance of the *HBB* genotype effects on the model was assessed using a likelihood ratio test that compared the above model to the same one but without the fixed effects of the *HBB* genotypes. Similar analyses were done for Fulani and Non-Fulani data separately.

**Table S2.** Lifetime table according to *HBB* genotype, Burkina Faso, 2007-8.

| **Ethnicity** | ***HBB*** | **Survey** | **Total (n)** | **Infection (n)** | **Lost (n)** | **Proportion not infected** | **95% CI** |
| --- | --- | --- | --- | --- | --- | --- | --- |
| Fulani | AA | 1 | 311 | 119 | 0 | 0.62 | 0.56-0.67 |
|  |  | 2 | 192 | 21 | 58 | 0.54 | 0.48-0.59 |
|  |  | 3 | 113 | 3 | 27 | 0.52 | 0.46-0.58 |
|  |  | 4 | 83 | 12 | 12 | 0.44 | 0.37-0.50 |
|  |  | 5 | 59 | 4 | 55 | 0.38 | 0.31-0.46 |
|  | AC | 1 | 53 | 21 | 0 | 0.61 | 0.46-0.72 |
|  |  | 2 | 32 | 3 | 9 | 0.54 | 0.39-0.66 |
|  |  | 3 | 20 | 1 | 5 | 0.51 | 0.36-0.64 |
|  |  | 4 | 14 | 4 | 1 | 0.36 | 0.20-0.51 |
|  |  | 5 | 9 | 0 | 9 | 0.36 | 0.20-0.51 |
|  | AS | 1 | 35 | 8 | 0 | 0.77 | 0.59-0.88 |
|  |  | 2 | 27 | 3 | 6 | 0.67 | 0.49-0.81 |
|  |  | 3 | 18 | 0 | 3 | 0.67 | 0.49-0.81 |
|  |  | 4 | 15 | 1 | 2 | 0.63 | 0.43-0.77 |
|  |  | 5 | 12 | 0 | 12 | 0.63 | 0.43-0.77 |
|  | SC | 1 | 2 | 1 | 0 | 0.50 | 0.01-0.91 |
|  |  | 2 | 1 | 0 | 1 | 0.50 | 0.01-0.91 |
| Non-Fulani | AA | 1 | 548 | 308 | 0 | 0.44 | 0.40-0.48 |
|  |  | 2 | 240 | 49 | 96 | 0.33 | 0.29-0.37 |
|  |  | 3 | 95 | 7 | 31 | 0.30 | 0.26-0.34 |
|  |  | 4 | 57 | 22 | 3 | 0.18 | 0.14-0.23 |
|  |  | 5 | 32 | 5 | 27 | 0.13 | 0.09-0.19 |
|  | AC | 1 | 165 | 87 | 0 | 0.47 | 0.39-0.55 |
|  |  | 2 | 78 | 19 | 23 | 0.34 | 0.26-0.41 |
|  |  | 3 | 36 | 4 | 10 | 0.29 | 0.22-0.37 |
|  |  | 4 | 22 | 6 | 4 | 0.21 | 0.13-0.29 |
|  |  | 5 | 12 | 5 | 7 | 0.08 | 0.03-0.18 |
|  | AS | 1 | 38 | 15 | 0 | 0.61 | 0.43-0.74 |
|  |  | 2 | 23 | 4 | 10 | 0.47 | 0.30-0.63 |
|  |  | 3 | 9 | 1 | 2 | 0.41 | 0.23-0.58 |
|  |  | 5 | 6 | 0 | 6 | 0.41 | 0.23-0.58 |
|  | CC | 1 | 5 | 3 | 0 | 0.40 | 0.05-0.75 |
|  |  | 2 | 2 | 0 | 1 | 0.40 | 0.05-0.75 |
|  |  | 5 | 1 | 0 | 1 | 0.40 | 0.05-0.75 |
|  | SC | 1 | 5 | 1 | 0 | 0.80 | 0.20-0.97 |
|  |  | 2 | 4 | 0 | 2 | 0.80 | 0.20-0.97 |
|  |  | 5 | 2 | 0 | 2 | 0.80 | 0.20-0.97 |

Abbreviations: *HBB*, *HBB* genotype; CI, Confidence Interval.

**Table S3**. Effect of HbS on susceptibility to *P. falciparum* asymptomatic infection by age-group and ethnicity, Burkina Faso, 2007-8.

| **a) Odds of infection** | | | |
| --- | --- | --- | --- |
| **Ethnicity** | **OR** | **95% CI** | **P-value** |
| **≤ 10 years old** | | | |
| Fulani | 0.71 | 0.08-6.59 | 0.764 |
| Non-Fulani ^a^ |  |  |  |
| Overall | na | na | na |
| **> 10 years old** | | | |
| Fulani | 0.13 | 0.02, 1.01 | 0.051 |
| Non-Fulani | 0.39 | 0.12, 1.26 | 0.117 |
| Overall | 0.28 | 0.11, 0.71 | 0.008 |
| **b) Number of infections** | | | |
| **Ethnicity** | **IRR** | **95% CI** | **P-value** |
| **≤ 10 years old** | | | |
| Fulani | 0.81 | 0.45, 1.45 | 0.477 |
| Non-Fulani | 1.02 | 0.61, 0.71 | 0.937 |
| Overall | 0.91 | 0.62, 1.35 | 0.651 |
| **> 10 years old** | | | |
| Fulani | 0.11 | 0.02, 0.79 | 0.028 |
| Non-Fulani | 0.68 | 0.41, 1.13 | 0.134 |
| Overall | 0.52 | 0.32, 0.85 | 0.010 |
| **c) Mean parasite count** | | | |
| **Ethnicity** | **OR** | **95% CI** | **P-value** |
| **≤ 10 years old** | | | |
| Fulani | 0.77 | 0.43, 1.36 | 0.369 |
| Non-Fulani | 1.11 | 0.60, 2.05 | 0.739 |
| Overall | 0.90 | 0.59, 1.36 | 0.619 |
| **> 10 years old** | | | |
| Fulani | 0.76 | 0.57, 1.00 | 0.051 |
| Non-Fulani | 0.71 | 0.45, 1.14 | 0.152 |
| Overall | 0.73 | 0.55, 0.98 | 0.036 |
| **d) Hazard of infection** | | | |
| **Ethnicity** | **HR** | **95% CI** | **P-value** |
| **< = 10 years old** | | | |
| Fulani | 0.88 | 0.44, 1.73 | 0.705 |
| Non-Fulani | 0.94 | 0.35, 2.54 | 0.910 |
| Overall | 0.90 | 0.52, 1.59 | 0.728 |
| **> 10 years old** | | | |
| Fulani | 0.28 | 0.09, 0.90 | 0.033 |
| Non-Fulani | 0.66 | 0.39, 1.09 | 0.106 |
| Overall | 0.54 | 0.34, 0.86 | 0.010 |

Abbreviations: HR, Hazard Ratio; IRR, Incidence Rate Ratio; OR, Odds Ratio; CI, Confidence Interval; *P*, P-value. Statistical methods: a) Odds of infection were compared by logistic regression; b) the numbers of infections were compared by Poisson regression; c) mean parasite counts were compared by linear regression and d) the cumulative probability of infection was compared by Poisson regression.

^a^ All infected.

**Supplementary methods.** Detection of *Plasmodium falciparum* in human DNA by Sequenom iPLEX genotyping (Agena Bioscience).

Alongside the genotyping of human SNPs undertaken as part of the MalariaGEN Consortial Project 2 (<http://www.malariagen.net/projects/cp2>), assays for 2 *P. falciparum* alleles were included in order to provide baseline information to validate *P. falciparum* parasitaemia measured by microscopy. All genotyping was performed using the Sequenom MassArray System (Agena Bioscience) that has been shown to be a useful tool to genotype malaria parasites [1].

The two assays measured alleles in the genes PfEBA175 and PfTRAP. These were chosen because they designed viable assays that fitted into the multiplexes of human SNPs for the above project. Assay design details are included in the Table below.

The sensitivity of each assay was assessed using a serial dilution of *P. falciparum* DNA in human DNA. *P. falciparum* DNA (3D7) was diluted in a pool of 30 human DNAs (CEPH individuals from the HapMap project). A stock sample of 3.2ng 3D7 + 98.8ng human DNA was prepared and diluted using a solution of 100ng/ul human DNA in a 4-fold series. Pooled human DNA alone was used as background control. Samples were plated for genotyping in replicates of 6 wells. All samples underwent PEP amplification [2] using 5ul of 1ng/ul DNA per reaction. Genotyping was undertaken on the PEP samples as described [1].

The dilution of 3D7 DNA in human DNA was transformed into genomes per PEP reaction where 1 genome of *P. falciparum* ~25fg; therefore 3.2ng *P.falciparum* DNA ~ 128,000 genomes.

The minimal signal intensity above background for the 2 assays fell between 0.48-1.95 genomes for the EBA175 assay and 1.95-7.81 for the TRAP assay. While the 2 assays showed different maximal signal intensities they both agree on a parasite detection cut-off ~2 genomes per 100ng human DNA (both reached a plateau intensity at ~32 genomes). This equates to a sensitivity of ~1 parasite per uL of human blood (using an average RBC count of 10^9^/ml and WBC count of 5x10^6^ per ml).

In the Burkina Faso study, the *P. falciparum* PCR assay was run on 1849 DNA samples obtained from 2 ml venous blood collected in EDTA tubes. The assay confirmed microscopic positivity and detected 6.1% samples (N=112) with possible sub-microscopic infection (microscopy negative and PCR positive).

References:

1. Manske M, Miotto O, Campino S, Auburn S, Almagro-Garcia J, Maslen G, O'Brien J, Djimde A, Doumbo O, Zongo I, Ouedraogo JB, Michon P, Mueller I, Siba P, Nzila A, Borrmann S, Kiara SM, Marsh K, Jiang H, Su XZ, Amaratunga C, Fairhurst R, Socheat D, Nosten F, Imwong M, White NJ, Sanders M, Anastasi E, Alcock D, Drury E, Oyola S, Quail MA, Turner DJ, Ruano-Rubio V, Jyothi D, Amenga-Etego L, Hubbart C, Jeffreys A, Rowlands K, Sutherland C, Roper C, Mangano V, Modiano D, Tan JC, Ferdig MT, Amambua-Ngwa A, Conway DJ, Takala-Harrison S, Plowe CV, Rayner JC, Rockett KA, Clark TG, Newbold CI, Berriman M, MacInnis B, Kwiatkowski DP. (2012). Analysis of Plasmodium falciparum diversity in natural infections by deep sequencing. Nature. 487:375-9.
2. Zhang L, Cui X, Schmitt K., Hubert R, Navidi W & Arnheim N. (1992). Whole genome amplification from a single cell: implications for genetic analysis. Proc. Natl. Acad. Sci. USA, 89, 5847-5851.

| **SNP_ID** | **PfTRAP H488Y** | **PfEBA175 V996G** |
| --- | --- | --- |
| Gene symbol | TRAP (PF13_0201) | EBA175 (MAL7P1.176) |
| Chromosome | 13 | 7 |
| Coordinate | 1465158 | 1361041 |
| 2nd-PCRP | ACGTTGGATGGAAACACGTCCACATGGTAG | ACGTTGGATGAGCAGTTATTGGGAGCAGTC |
| 1st-PCRP | ACGTTGGATGTGGCTTTTCATGTTCTTCCC | ACGTTGGATGCGTACATCATCAGATAAAGG |
| AMP_LEN | 119 | 119 |
| UEP_DIR | R | R |
| UEP_MASS | 6946.5 | 7977.2 |
| UEP_SEQ | TTCATGTTCTTCCCTTTCAGGAT | CATCATCAGATAAAGGTTTAGAATTC |
| EXT1_CALL | **T** | G |
| EXT1_MASS | 7217.7 | 8224.4 |
| EXT1_SEQ | TTCATGTTCTTCCCTTTCAGGATA | CATCATCAGATAAAGGTTTAGAATTCC |
| EXT2_CALL | **C** | T |
| EXT2_MASS | 7233.7 | 8248.4 |
| EXT2_SEQ | TTCATGTTCTTCCCTTTCAGGATG | CATCATCAGATAAAGGTTTAGAATTCA |

Table. Sequenom iPLEX assay designs for 2 *P. falciparum* alleles. Chromosome and coordinates are taken from the *P. falciparum* reference genome version 3. Gene Symbols show both the common name and the standardised name from the ApiLoc database (http://apiloc.biochem.unimelb.edu.au/apiloc/apiloc).

UEP: universal extension primer, Mass in Daltons, UEP_DIR: Assay design direction relative to the input sequence which here was on the forward strand; Extension primer sequences are therefore with respect to the direction of assay design. AMP_LEN is the first round amplicon length that includes the first-round Sequenom primer 5' tails (Highlighted) that are part of the assay design process.
